# Genome-wide identification of *Cymbidium sinense* WRKY gene family and the importance of its Group III members in response to abiotic stress

**DOI:** 10.3389/fpls.2022.969010

**Published:** 2022-07-28

**Authors:** Yong-Lu Wei, Jian-Peng Jin, Di Liang, Jie Gao, Jie Li, Qi Xie, Chu-Qiao Lu, Feng-Xi Yang, Gen-Fa Zhu

**Affiliations:** Guangdong Key Laboratory of Ornamental Plant Germplasm Innovation and Utilization, Environmental Horticulture Research Institute, Guangdong Academy of Agricultural Sciences, Guangzhou, China

**Keywords:** *Cymbidium sinense*, genome-wide, WRKY, expression pattern, abiotic stress

## Abstract

Transcription factors (TFs) of the WRKY family play pivotal roles in defense responses and secondary metabolism of plants. Although WRKY TFs are well documented in numerous plant species, no study has performed a genome-wide investigation of the WRKY gene family in *Cymbidium sinense*. In the present work, we found 64 *C. sinense* WRKY (CsWRKY) TFs, and they were further divided into eight subgroups. Chromosomal distribution of *CsWRKYs* revealed that the majority of these genes were localized on 16 chromosomes, especially on Chromosome 2. Syntenic analysis implied that 13 (20.31%) genes were derived from segmental duplication events, and 17 orthologous gene pairs were identified between *Arabidopsis thaliana WRKY* (*AtWRKY*) and *CsWRKY* genes. Moreover, 55 of the 64 *CsWRKYs* were detectable in different plant tissues in response to exposure to plant hormones. Among them, Group III members were strongly induced in response to various hormone treatments, indicating their potential essential roles in hormone signaling. We subsequently analyzed the function of *CsWRKY18* in Group III. The *CsWRKY18* was localized in the nucleus. The constitutive expression of *CsWRKY18* in Arabidopsis led to enhanced sensitivity to ABA-mediated seed germination and root growth and elevated plant tolerance to abiotic stress within the ABA-dependent pathway. Overall, our study represented the first genome-wide characterization and functional analysis of WRKY TFs in *C. sinense*, which could provide useful clues about the evolution and functional description of *CsWRKY* genes.

## Introduction

As one of the most prominent flowering plant families, Orchidaceae include 801 genera and more than 30,000 specie (Brown, 2005). In the orchid family, Cymbidium is widely fostered in East Asia, such as China, Japan, and Korea, and Southeast Asia (Liu et al., 2006). Cymbidium has become very prevalent in China, and it is highly desirable in traditional flower markets because of its beauty, fragrant flowers, elegant and upright leaves. However, functional genomics investigations and gene identification of valuable horticultural traits are significantly restricted because of the polyploid genomes and long juvenile phases of the genus Cymbidium. Therefore, it is essential for discovery of functional genes in Cymbidium research. In recent years, next-generation sequencing (NGS) technologies have rapidly developed, offering formidable approaches for high-throughput sequence determination (Leitch et al., 2009; Liu et al., 2012). Furthermore, as RNA-Seq decrease in cost, promoting the identification of new genes by obtaining massive amounts of sequence data with enormous depth and coverage. As a result, a large number of critical regulators related to important agronomic traits and environmental adaptation have been identified in orchid species, including Apostasia (Zhang et al., 2017, 2021a), Cymbidium (Ai et al., 2021; Yang et al., 2021), Dendrobium (Niu et al., 2021; Zhang et al., 2021b), Gastrodia (Xu et al., 2021) Phalaenopsis (Cai et al., 2015; Chao et al., 2018), and Vanilla (Hasing et al., 2020).

The transcription factors (TFs) are critical in controlling plant development and stress response. WRKY, one of the largest families of higher plant TFs, plays essential roles in pathogen defense, abiotic cues, phytohormone signaling, and management of plant development and secondary metabolism. The WRKY domain (WD) is the essential characteristic of the WRKY protein, which is ~60 amino acids in length and consists of the highly conserved signature WRKYGQK, followed by a C2H2-or C2HC-type of zinc-finger motif. The high binding affinity of WRKY TFs to the consensus W-box cis-elements requires both heptapeptide sequence and zinc-finger motif (Maeo et al., 2001). Given these features of the WD, its family members are categorized into three groups (Eulgem et al., 2000). The WRKY Group II can be further divided into five subgroups (IIa, IIb, IIc, IId, and IIe) according to their primary amino acid sequences (Chen et al., 2017). In addition, WRKY preferentially binds to a markedly conservative DNA motif named the W box (T/CTGACC/T; Rushton et al., 2010). Since the first WRKY TF (SPF1) is cloned from sweet potato (*Ipomoea batatas*; Ishiguro and Nakamura, 1994), an increasing number of WRKY family genes are identified in several species, including Arabidopsis (75; Wu et al., 2005), barley (103; Kan et al., 2021), flax (102; Yuan et al., 2021), ginseng (137; Di et al., 2021), Gossypium (109; Ding et al., 2015), grape (59; Wang et al., 2015), Musa (147; Goel et al., 2016), maize (125; Hu et al., 2021), Taxus (61; Zhang et al., 2018), rice (109; Okay et al., 2014), walnut (103; Hao et al., 2021), and wheat (124; Ye et al., 2021).

In the present study, we analyzed the genome sequences of *Cymbidium sinense*, a very famous traditional orchid plant in China and Southeast Asia, and identified 64 *CsWRKY* proteins. Computational analysis was conducted to assess their physicochemical properties, including conserved domain, phylogenetic relationship, motif composition, functional annotation, and protein–protein interaction (PPI). In addition, the expression profiling of *CsWRKY* genes in various plant tissues in response to hormone treatments abscisic acid (ABA), gibberellic acid, salicylic acid, and methyl jasmonate (MeJA) indicated that most *CsWRKY* genes were responsive to various plant hormones, and Group III genes appeared to be more actively expressed to cope with the stress response. Collectively, our current findings provided valuable insights into the understanding of WRKY TFs in orchid plants and identified many candidate regulators involved in multiple hormone signaling.

## Results

### Identification and analysis of WRKY genes in *Cymbidium sinense*

A total of 64 WRKY TFs were found through the *C. sinense* genomic database (Supplementary Table S2). Each WRKY gene was consistently named *CsWRKY1*-*CsWRKY64* based on its chromosomal location. The characteristics were analyzed, including the genome location, length of the open reading frame (ORF), and basic information of their encoded proteins, including length, molecular weight (MW), and isoelectric point (pI). Supplementary Table S2 shows that the length of CsWRKY proteins ranged from 67 (*CsWRKY38*) to 676 (*CsWRKY61*) amino acids, and their average length was 317 residues. The pI ranged from 4.84 (*CsWRKY46*) to 10.00 (*CsWRKY62*), and the MW ranged from 5,929.61 Da (*CsWRKY12*) to 7,4025.70 Da (*CsWRKY61*).

### Classification of *Cymbidium sinense* proteins

**Figure 1:**
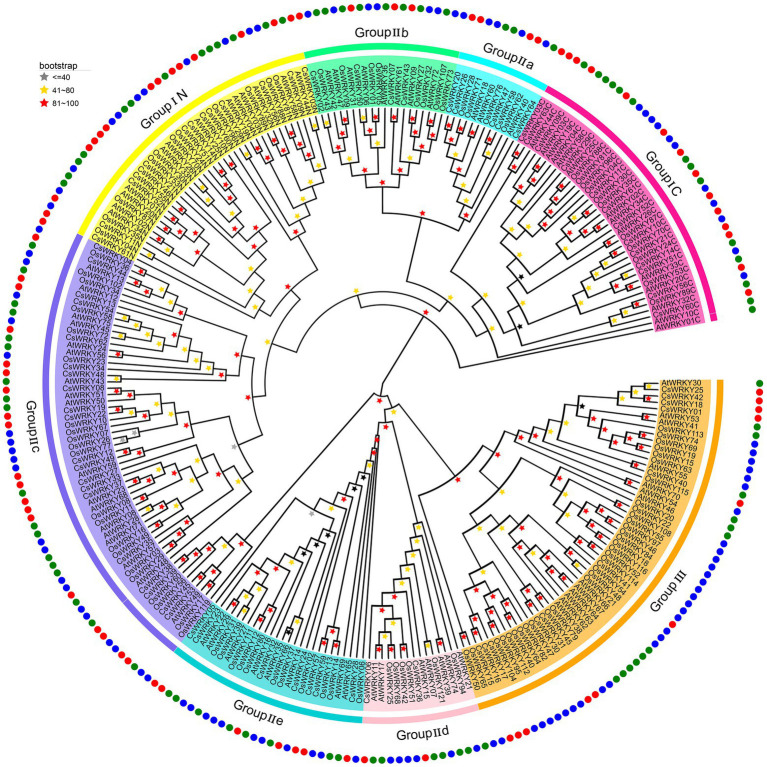
Phylogenetic tree of WRKY genes in *Cymbidium sinense* (Cs), *Arabidopsis thaliana* (At), and *Oryza sativa* (Os). An individual group (I–III) of ancestral relationships were presented in different colors.

To explore the phylogenetic relationship among *CsWRKY* proteins, 275 conserved WDs, including 114 *OsWRKY* proteins from rice (*O. sativa* japonica) and 85 *AtWRKY* proteins from Arabidopsis, were extracted to construct the evolutionary tree using the maximum likelihood (ML) method. As shown in the evolutionary tree (Figure 1), the 64 *CsWRKY* proteins were categorized into three main groups (I, II, and III), and Group II proteins were further sorted into five subgroups (IIa, IIb, IIc, IId, and IIe). The *CsWRKY* TFs had two standard motifs, including a WD and a zinc-finger-like domain. The former, typically the WRKYGQK sequence, could combine with the W box cis-element to trigger the expressions of their downstream genes. Besides the WRKYGQK sequence, three variants, WKKYGQK (*CsWRKY47*) in Group IIa, WRKYGKK (*CsWRKY08*, *19*, and *22*) in Group IIb, and WRKYGRK (*CsWRKY49*, and 62) in Group IIc were also revealed, respectively. The latter, the zinc-finger-like domain, also had two types, namely C2H2 and C2HC.

It is worth noting that Group I was composed of 15 *CsWRKY* proteins, 11 of which contained two WDs. The remaining *CsWRKY63* had a single intact WD in its C terminus, and the other three members (*CsWRKY03*, *46*, and *55*) had a single WD at the N terminus (Supplementary Table S2; Supplementary Figure S1), implying that they most likely experienced domain loss or acquisition events in the evolutionary process (Ross et al., 2007). In addition, the zinc-finger motifs of the *CsWRKYs* in Group I belonged to the C2H2 type with a C-X4-C-X22–23-H-X1-H motif (Supplementary Table S2). There were 38 members in Group II, while 75 contained the motif of C-X4–5-6-C-X23–25-29-H-X1-H, and three members, *CsWRKY01*, *38*, and *44*, lacked a typical zinc-finger-like motif. According to their phylogenetic relationship, all 38 Group II members were further categorized into five subgroups as follows: Group IIa (five proteins), Group IIb (four proteins), Group IIc (20 proteins), Group IId (two proteins), and Group IIe (seven proteins). The zinc-finger motifs of Group III members (11) belonged to the C2HC type, with the C-X7-C-X23–24-26-H-X1-C motif (Supplementary Table S2), except for CsWRKY17 and 41 only containing fragmentary WRKY structure.

### Analyses of chromosomal location, gene duplication, and genome synteny

**Figure 2:**
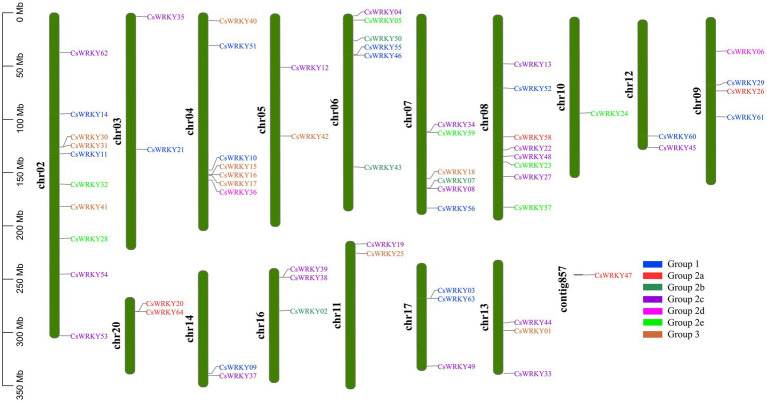
The chromosomal distribution and positioning of *CsWRKYs* across all seven chromosomes of *C. sinense*. A total of 16 chromosomes and one contig with varying lengths are shown in Mb (million base pair) scale on the left, where individual chromosomes (bars) are labeled with respective *CsWRKY* genes. The subfamily members are presented in various colors.

A total of 64 candidate *CsWRKY* genes were mapped to 16 of the 20 chromosomes of *C. sinense* with an irregular arrangement (Figure 2). Chromosome 2 harbored the most significant number of *CsWRKYs* (10 genes), followed by chromosome 8 (eight genes). With only one gene, the least number of *CsWRKYs* was found on Chromosome 10. Three chromosomes contained the members from all three groups, 10 chromosomes contained the members from two groups, and the other three only had the members from one group.

To further assess the origin and evolution of the *CsWRKY* gene family, we constructed two comparative syntenic maps between *C. sinense* and *A. thaliana*, *O. sativa*, *V. vinifera*, *M. cuminata*, or *Z. mays* at the genome-wide level. Supplementary Figure S2 shows that we finally identified *27*, *28*, and *29* orthologous gene pairs between *C. sinense* and *O. sativae*, *V. vinifera*, or *M. cuminata*, respectively. However, only 12 and 19 orthologous gene pairs between *C. sinense* and *Z. mays* or *A. thaliana*, were identified, respectively. More details about these orthologous gene pairs are given in Supplementary Table S5. There were far more orthologous genes between *C. sinense* and *O. sativae* than those between *C. sinense* and *A. thaliana*, which probably resulted from the nearer phylogenetic relationship between *C. sinense* and *O. sativae*.

To assess the selection pressure on various duplicated WRKY genes, we calculated the Ka and Ks substitution rates and the Ka/Ks ratios for each repeat *CsWRKY* gene pair. Typically, a Ka/Ks ratio of 1, >1, or <1 indicates neutral selection, adaptive evolution with positive selection, and negative or purifying selection, respectively (Nekrutenko et al., 2002). The Ka/Ks ratio for all seven segmentally duplicated gene pairs was <1 (Supplementary Table S4; Supplementary Figure S3), showing the high conservation of *CsWRKY* genes during evolution.

### Gene structure and conserved motif analysis of *CsWRKY* genes

**Figure 3:**
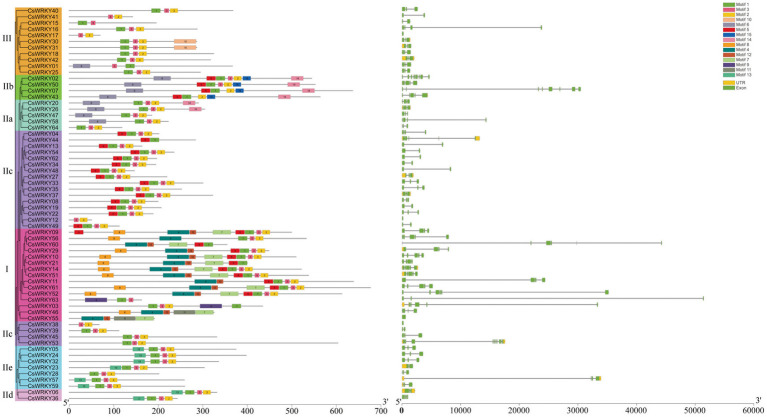
Phylogenetic relationships, gene structure, and architecture of conserved protein motifs in WRKY genes from *C. sinense*.

To gain more in-depth insight into the development of the WRKY gene family in *C. sinense*, we mapped the genetic structure of each *CsWRKY* gene. Based on the full-length *CsWRKY* proteins (Figure 3), we constructed a phylogenetic tree to clarify the gene structure and conserved motifs better. The number of introns in *CsWRKY* genes ranged from 0 to 10, similar to rice from 0 to 8 (Xie et al., 2005). Most *CsWRKY* genes harbored one to four introns, with 23 members containing two introns, 13 containing three introns, 10 containing one intron, and nine containing four introns. The other two *CsWRKY* genes contained five introns, and three *CsWRKY* genes contained seven, eight, and 10 introns. Additionally, the remaining four *CsWRKY* genes contained no intron. Figure 3 reveals that most *CsWRKY* genes within the same group or subgroup had a similar gene organization, showing the functional similarity among members.

Moreover, we identified 15 conserved motifs within proteins by the MEME program. MEME motif study showed that each *CsWRKY* protein had its specific conserved motifs (Supplementary Table S3). Figure 3 depicts that the WRKY family members with similar motif structures could be categorized into the same group. Almost all *CsWRKY*s had the conserved heptapeptides WRKYGQK (Motif 1 or Motif 4) and harbored at least one motif. Besides, Motif 2 was composed of the C2H2 motif, while Motif 12 consisted of the C2HC motif. Furthermore, the conserved motifs were explicitly present in various groups. For instance, Group IIa members harbored four conserved motifs (Motifs 1, 2, 3, and 6), Group IIb members contained five conserved motifs (Motifs 1, 2, 5, 6, and 15), and 14 Group IIc members contained four conserved motifs (Motifs 1, 2, 3, and 5). Clearly, a few motifs existed in one or more groups and subgroups. For example, Motifs 4, 8, 7, and 12 existed in Group I members, and Motif 10 existed only in Group III, while Motif 15 was mainly present in Group IIb. This result suggested that these groups were endowed various functions during evolution. However, even if these members belong to the same clade, the functions of family members vary greatly (Yang et al., 2019). These WRKY proteins may have different functions though classified in the same group. Despite CsWRKY28 belonged to group IIe, it had the similar conserved motifs with the group IIc.

### The cis-elements in the promoters of *Cymbidium sinense* WRKY genes

Cis-elements in promoter region are important for gene expression (Rombauts et al., 1999). To verify the potential function of *CsWRKY* genes in response to abiotic stress, we extracted the 2,000-bp promoter sequences of the *CsWRKY* genes and analyzed them for cis-elements using the PlantCARE database. Supplementary Figure S4 depicts that 20 types of cis-acting elements related to stresses and phytohormone responses were identified in the promoters of *CsWRKY* genes, including four defense-and stress-responsive elements (W box and TC-rich repeats) and seven hormone-related elements (ABRE, AuxRE, As-1, TGA-element, CGTCA-motif, and TGACG-motif). Supplementary Figure S4 shows that MYC and MYB were the most numerous elements in the promoter regions of all 64 *CsWRKYs*, with 61 genes containing these two elements. Box 4 and G box were two types of light-response elements, ARE was involved in anaerobic induction, and ABRE (ABA-responsive elements), CGTCA-motif, and TGACG-motif (MeJA-responsive elements) also appeared frequently in promoter regions of *CsWRKY* genes, which were found in 59, 49, 49, 47, 42, and 42 promoters, respectively. Many W-boxes were identified in 37 *CsWRKY* gene promoters, indicating that these genes were modulated by other WRKY TFs or themselves. LTR and MBS elements that respond to low temperature and drought stresses were identified in the promoters of 17 and 28 *CsWRKY* genes, respectively. Moreover, 25 promoters showed a TC-rich repeats element (cis-acting element participated in defense and stress response), and only 15 promoters had a GARE-motif (gibberellin-responsive element). Thus, the cis-element analysis showed that the expressions of *CsWRKY* genes in *C. sinense* might be related to various environmental factors.

### Interaction analysis of specific *CsWRKY* proteins

A PPI network of *CsWRKY* proteins was established using STRING 10.5 software based on the Arabidopsis association model. Our results showed that there were close interaction networks among 43 *CsWRKYs* (Supplementary Figure S5). Among them, 14 proteins belonged to Group I, 22 proteins belonged to Group II, and seven proteins belonged to Group III. The extensive WRKY-WRKY interactions manifested a mechanism for functional cooperation and antagonism among WRKY proteins for dynamic regulation of target genes in *C. sinense*. In addition, *CsWRKY20*, *CsWRKY26*, *CsWRKY47*, *CsWRKY58*, *and CsWRKY64* belonging to Group IIa, *CsWRKY10*, *CsWRKY11*, *CsWRKY14*, *CsWRKY21*, and *CsWRKY51* belonging to Group I, and *CsWRKY01*, *CsWRKY25*, *CsWRKY42* and *CsWRKY15* belonging to Group III were also involved in a more robust interaction network with other proteins. For instance, MAP kinase 4 (MPK4), functioning as a regulator of pathogen defense responses (Rasmussen et al., 2012; Bazin et al., 2020), and SIB1 are negative regulators of ABA-mediated leaf senescence (Zhang et al., 2022a), indicating that these genes played a kernel role in the biotic and abiotic stress response.

### Analysis of expression profiles of *Cymbidium sinense* WRKY genes in different tissues

**Figure 4:**
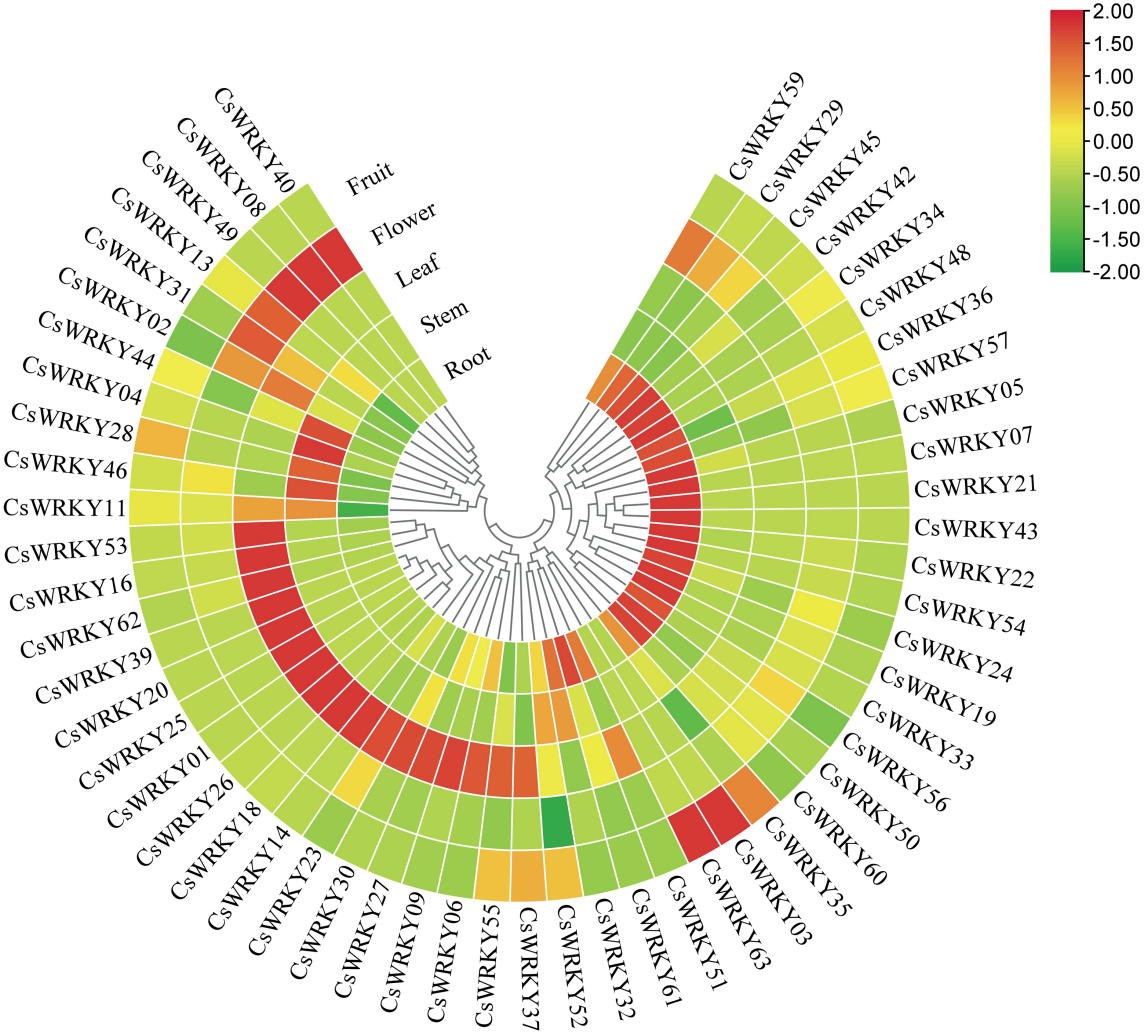
Heat map representation and hierarchical clustering of the *CsWRKY* gene expression profiles in five tissues.

Based on RNA-seq and expression profiling analysis, 55 *CsWRKYs* were detectable in different plant tissues (Figure 4; Supplementary Table S6). However, the other nine transcripts (*CsWRKY10*, *12*, *15*, *17*, *38*, *41*, *47*, *59*, and *64*) were not detected, showing that these genes might be pseudogenes or have particular temporal and spatial expression patterns. Most of the 55 *CsWRKY* transcripts investigated were expressed in all tissues with low expression levels, suggesting that these TFs worked with other proteins synergistically or interactively during plant growth and development. Moreover, many genes had preferential expression in various tissues. For example, 19 *CsWRKY* transcripts (*CsWRKY21*, *55*, *56*, *60*, and *61* from Group I, CsWRKY*07*, *43*, and *50* from Group IIb, *CsWRKY19*, *22*, *33*, *34*, *48*, and *54* from Group IIc, *CsWRKY25* from Group IId, *CsWRKY05*, *24*, and *57* from Group IIe, and *CsWRKY42* from Group III) exhibited high expression levels in root particularly. By contrast, 2, 10, 3, and 2 exhibited the highest transcript abundances in the stem, leaf, flower, and fruit, respectively. These genes might play special roles in specific tissues. Additionally, several gene pairs with a close relationship, such as *CsWRKY03/63* and *CsWRKY19/33*, indicated similar expression profiles, suggesting that the functionality of these genes was redundant.

### Expression profiles of *Cymbidium sinense* WRKY genes with hormone treatment

WRKY are widely involved in plant abiotic stress regulation (Jiang et al., 2017). To further certify the hormone response of *CsWRKY* genes, quantitative real-time PCR (qRT-PCR) was accomplished for 21 randomly chosen *CsWRKY* genes, including four Group I members, 11 Group II members, and six Group III members. We first optimized the treatment conditions, and the seedling leaves were sampled after exposure to 100 μmol GA, ABA, SA, IAA, JA, or ACC for 2 h.

**Figure 5:**
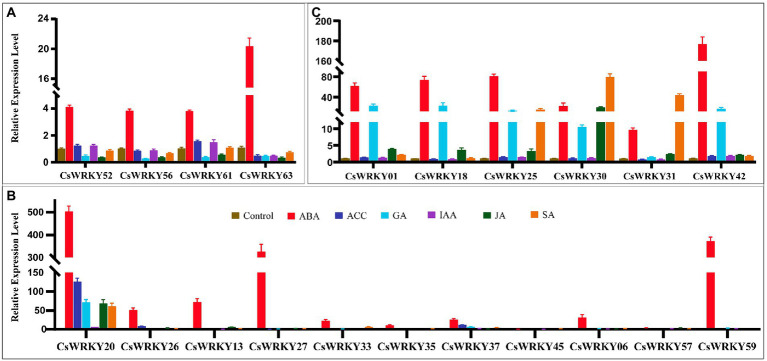
Expression patterns of 21 selected *CsWRKY* genes in *C. sinense* leaves under ABA, ACC, GA, IAA, JA, and SA stresses (**A**. Group I, **B**. Group II, and **C**. Group III). qRT-PCR data were normalized using the Cymbidium actin gene. X-axes represent various treatments (CK, normal condition). Different genes and y-axes are scales of relative expression level. Error bars result from three biological replicates.

Figure 5 shows that the expressions of *CsWRKY01*, *CsWRKY18*, *CsWRKY20*, *CsWRKY25*, *CsWRKY30*, *CsWRKY31*, *CsWRKY37*, and *CsWRKY42* were significantly up-regulated by over 5-fold under the GA treatment (Figures 5A,B). Under the ABA treatment, all of *CsWRKYs* were significantly induced, except that *CsWRKY45* was significantly down-regulated (Figure 5). Under the SA treatment, *CsWRKY20*, *CsWRKY25*, *CsWRKY30*, and *CsWRKY31* were significantly induced by over 15-fold (Figures 5B,C). The expression of *CsWRKY20* was up-regulated by over 5-fold under the IAA treatment, which was induced by over 6-fold under the JA treatment and over 125-fold under the ACC treatment (Figure 5B). More interestingly, Group III genes sensitive to responses to various hormones played important roles in responses to abiotic stress in *C. sinense*.

### Subcellular localization of *CsWRKY18*

**Figure 6:**
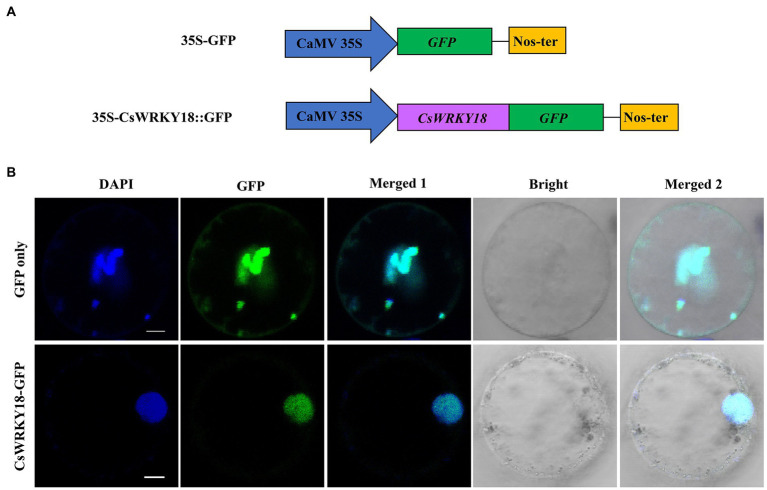
Subcellular localization of Group III *CsWRKY18* protein. **(A)** Schematic illustration of the constructs. **(B)** Subcellular location of free GFP and *CsWRKY18*-GFP protein in *C. sinense* leaves.

Since subcellular location information can provide some clues for protein function research, we used the online software Wolf PSORT to predict the subcellular locations of *CsWRKY* proteins in this study (Supplementary Table S7). Almost all CsWRKY proteins were mainly located in the nucleus, except for seven genes located in various organelles, such as cytoplasm, chloroplast, mitochondria, and peroxisome. *CsWRKY18* protein was chosen for subcellular localization verification. The full-length cDNA without the termination codon was fused in-frame to the 5′-end of the GFP gene under the control of the CaMV 35S promoter (Figure 6A). The recombined vector pAN580-*CsWRKY18*-GFP was transfected into leaf base protoplasts of Cymbidium. Confocal microscopy revealed that the fusion protein *CsWRKY18*-GFP was detected specifically in the nucleus, while GFP control displayed ubiquitous distribution in the whole cell. Furthermore, DAPI (4′,6-diamidino-2-phenylindole) staining showed that CsWRKY was clearly localized to the nucleus (Figure 6B).

### Overexpression of the *CsWRKY18* gene increases ABA sensitivity

**Figure 7:**
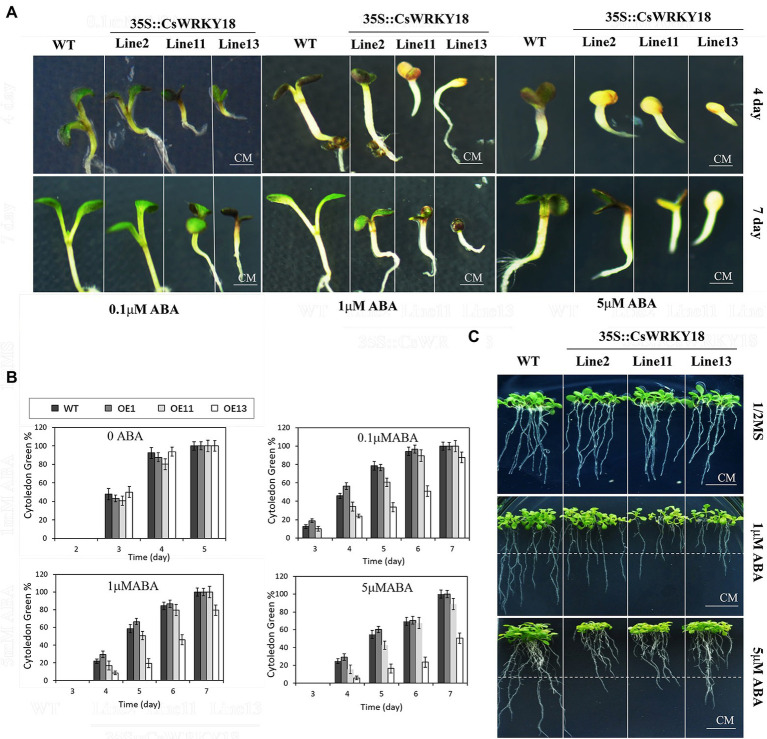
35S::*CsWRKY18* seedlings development under ABA treatment. **(A)** Seedling development of wild-type and 35S::*CsWRKY18* treated with 0.1, 1, and 5 μM ABA. **(B)** Cytoledon green ratio of seedlings under ABA treatment. **(C)** Root elongation of wild-type and 35S::*CsWRKY1*8.

Figure 7A shows that under normal conditions, no significant difference in germination between WT plants and three transgenic lines was found. However, when 5 μM exogenous ABA was given, the transgenic *Arabidopsis* lines showed dramatically lower cotyledon greening rates than WT plants. Moreover, during the post-germinative growth stage, 4-day-old seedlings grown on 0.5× MS medium were transferred to vertical agar plates containing 1 μM or 5 μM ABA. We found that the root growths of WT and transgenic lines were both markedly suppressed. However, the root lengths of transgenic plants were remarkably shorter compared with WT plants (Figure. 7C).

### Overexpression of *CsWRKY18* enhances plant tolerance to abiotic stress

**Figure 8:**
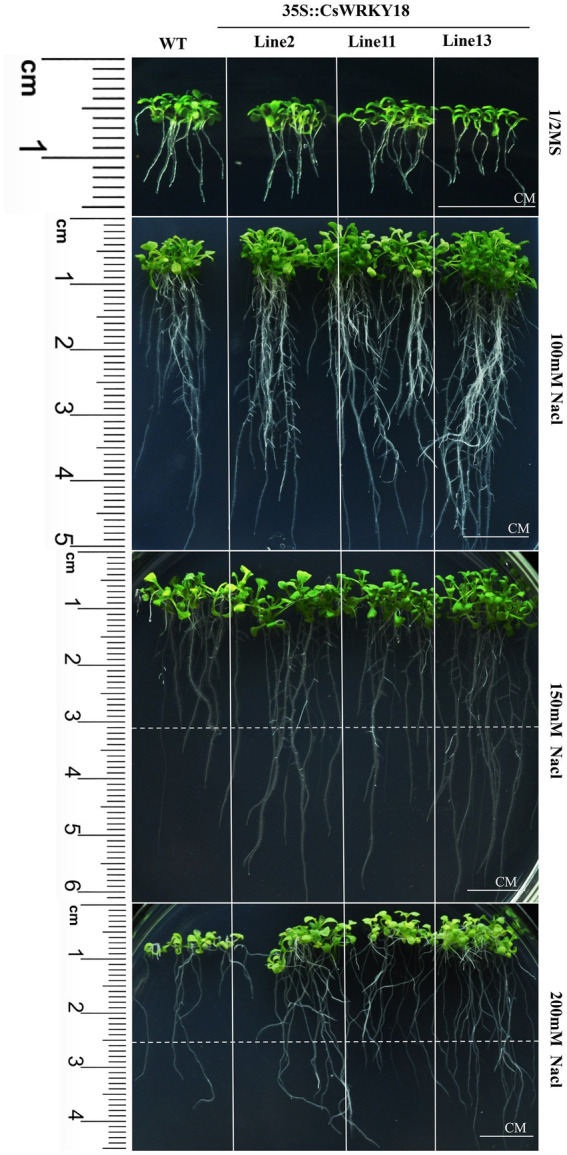
Root elongation of wild-type and 35S::*CsWRKY18* under NaCl treatment in 100, 150, and 200 mM.

We further compared the root morphology of the WT and transgenic plants grown on 1/2 MS medium containing 0 mM, 100 mM, 150 mM, and 200 mM NaCl (Figure 8). Three transgenic lines exhibited longer roots compared with WT plants growing on all culture medium (Figure 8). In parallel, leaves of 4-week-old seedlings were treated with 200 mg/L PEG6000. After 12 h, dehydration and shrinkage of the WT leaves were observed. In contrast, the leaves of transgenic plants remained fresh and upright (Supplementary Figure S6).

To explore the mechanism of *CsWRKY18* in response to salt and osmotic stress, Tu, a well-known ABA biosynthesis inhibitor, was applied. When 0.75 mM Tu was supplemented to a medium containing 150 mM NaCl buffer, the salt and osmotic tolerance phenotypes in the transgenic lines were obviously weakened (Supplementary Figure S7). These results suggested that the *CsWRKY18* transgenic plants were tolerant to salt and osmotic stress, indicating a potential role of *CsWRKY18* in the ABA pathway.

## Materials and methods

### Plant materials and treatment

In the present study, the plants of *C. sinense’* Dharma’ were grown in a greenhouse under controlled conditions (26°C/23°C, day/night, 16 h/8 h light/dark photoperiod with relative humidity 80%) at Environmental Horticulture Research Institute, Guangdong Academy of Agricultural Sciences (China). For tissue/organ-specific expression analysis, various plant parts, such as roots, leaves, stems, and flowers, were harvested from a 2-year-old plant. For expression in fruit tissues, 1/2/3/cm, mature green fruits, breaker fruits, and 10-day breaker fruits were harvested. For phytohormone treatment, 2-year-old plants in flowerpots were used, and each group (*n* = 3) was sprayed with 100 μM ABA, gibberellin (GA3), auxin (IAA), and MeJA. The samples were collected at 0, 0.5, 1, 2, 3, and 4 h after exposure from nine individual plants. For different organ samples, roots, stems, and leaves were collected from three plants grown for 45 days. All materials were immediately frozen in liquid nitrogen and preserved at −80°C for further analysis of WRKY genes. Considering the sensitive response of Group III to hormone signals, we randomly selected *CsWRKY18* for further functional analysis. We generated transgenic *A. thaliana* plants overexpressing the *CsWRKY18* gene under the CaMV 35S promoter, according to the general method (Clough and Bent, 1998; Feng et al., 2021) and we established 23 homozygous transgenic lines using hygromycin selection and PCR. Three T2-independent lines, L2, L11, and L13, were chosen for further phenotypic analysis according to the PCR analysis (Figure 7B). Seeds from transgenic and WT plants were incubated for germination on 1/2 MS medium supplemented with 1 μM ABA.

### Identification of WRKY genes from *Cymbidium sinense*

The genome sequences of *C. sinense*, which were sampled in Guangzhou, Guangdong Province, China, were established by our lab (Yang et al., 2021), and the HMM file (PF03106) of the WD was obtained from the Pfam database.^1^ To comprehensively identify the *CsWRKYs*, HMMER 3.0^2^ software was adopted to search against the *C. sinense* protein sequences with default parameters (*E*-value cut-off <1E-5). The incorrect and redundant predicted sequences were manually removed, and then all putative *CsWRKY* genes were further verified using NCBI’s Conserved Domain Database.^3^ The MW and PI were evaluated using ExPASy-ProtParam online software.^4^ Meanwhile, the protein sequences of *A. thaliana* and *O. sativa* were downloaded from TAIR^5^ and Rice Genome Annotation Project,^6^ respectively.

### Phylogenetic analysis of *Cymbidium sinense* WRKY TFs

Multiple sequence alignment of the WDs and full-length proteins was conducted by CLUSTAL (gap opening penalty: 10 and gap extension penalty: 0.2; Larkin et al., 2007). The phylogenetic tree was reconstructed from 1,000 ultrafast bootstrap ML tree replicates using IQ-TREE v1.6.12 (Nguyen et al., 2015) with best-fit model selection (PMB + I + G4) by ModelFinder (Kalyaanamoorthy et al., 2017). The ML phylogenetic tree was visualized using Evolview v3 (Subramanian et al., 2019).

### Characterization of the structure and motif of *CsWRKYs*

The available information of exons and introns was retrieved from the *C. sinense* genome sequences and then visualized by the TBtools v1.098725 (Chen et al., 2020) with coding sequences and genomic sequences. The MW and pI of the putative WRKY proteins were calculated by the ExPASy proteomics server.^7^ The motifs of each deduced *CsWRKY* protein were analyzed by MEME suite software^8^ (Bailey et al., 2009) with parameters as follows: the maximum number of motifs, 10. The upstream 2-Kb sequences of WRKY genes were extracted to determine the cis-elements by using the PlantCARE database.^9^

### Comparative genome synteny analysis

The alignment was performed by LASTZ using CDS sequences of *C. sinense* and five representative species (*A. thaliana*, *Oryza sativa*, *Vitis vinifera*, *Musa acuminate*, and *Zea mays*). The syntenic block map was established by MCscan with cscore = 0.7. Each orthologous gene pair was then further investigated with PAL2NAL^10^ (Suyama et al., 2006) to determine the Ks (synonymous substitution rate) and Ka (non-synonymous substitution rate).

### Transcriptomic data sets to analyze the expression patterns of WRKYs

To evaluate gene expression profiles of *CsWRKYs*, the expression patterns of *CsWRKY* genes in five different tissues were identified, such as roots, stems, leaves, flowers, and fruits. The transcriptome data of all tissues were came from our previous report (Yang et al., 2021). The expression values were calculated by log2 (FPKM) and displayed as a heat map generated using TBtools.

### Subcellular localization of *CsWRKY* proteins

The subcellular localization was predicted using the online software WOLF PSORT.^11^

### RNA extraction, cDNA synthesis, and qRT-PCR analysis

The above leaf samples were ground into a fine powder. Total RNA of these samples was extracted using Trizol Reagent (Invitrogen), and then 1 μg RNA was used to synthesize cDNA in a 20-μl reaction system according to the instructions in PrimeScript™ RT reagent Kit with gDNA Eraser (TAKARA). qRT-PCR was carried out on a Bio-Rad iCycler Real-Time PCR Detection System (United States) by TaKaRa SYBR Premix Ex Taq™ (Tli RNaseH Plus) with three biological replications. Briefly, the reaction was conducted in a 20-μl reaction system containing 10 μl SYBR Premix (2×), 1 μl cDNA, 1 μl sense and anti-sense primer (10 μM), and 7 μl ddH_2_O. After an initial denaturation step at 95°C for 1 min, the amplifications were carried out with 40 cycles at a melting temperature of 95°C for 10 s, an annealing temperature of 56°C for 30 s, and an extension temperature of 72°C for 30 s. Primer pairs were designed using Primer Premier 5.0, and the NCBI blast program was used to identify the specificity of all primers (Supplementary Table S1). The β-actin gene (Mol013347) in *C.sinense* was used as an internal reference, and the relative expression levels were calculated by the 2^−ΔΔCT^ method (Livak and Schmittgen, 2001).

### CsWRKY PPI analysis

A functional protein association network was constructed in the STRING program based on the Arabidopsis association model with the confidence parameter of 0.15 and the number of interactions of 5.

## Discussion

Genes of the WRKY superfamily, one of the largest groups of TFs in plants, are also present in other eukaryotic lineages, such as flagellated protozoan (*Giardia lamblia*) and soil-living amoeba-like slime mold (*Dictyostelium discoideum*; Zhang and Wang, 2005; Phukan et al., 2016). WRKY family plays a vital functional Progress in gene identification and functional evaluation of WRKY family in plant germination to senescence or seed production in the whole plant’s life cycle. WRKY TFs can enhance tolerance against heat, freezing, drought, salt, cadmium stress, UV radiation, and disease-causing organisms in different crop species, including Arabidopsis, Chrysanthemum, cucumber, peanut, pepper, rice, tobacco, tomato, and wheat (Dang et al., 2013; Liang et al., 2017; Chakraborty et al., 2018; Gao et al., 2018; Han et al., 2018, 2019; Hong et al., 2018; Khan et al., 2018; Kiranmai et al., 2018; Liu et al., 2018; Luan et al., 2019; Hussain et al., 2021).

However, compared with other crops, knowledge of the WRKY gene family has remained relatively scarce in the orchid field due to the relatively backward release of a complete and high-quality genome. In the present study, we identified 64 WRKY genes from the *C. sinense* genome. All *CsWRKY* were also classified into seven groups (groups I, IIa, IIb, IIc, IId, IIe, and III). Possibly since the green alga *Chlamydomonas reinhardtii* contains only one Group I WRKY gene (Zhang and Wang, 2005), Group I WRKY genes are considered the ancestors of other WRKY genes. In *C. sinense*, as many monocotyledonous species occur domain loss events (Ross et al., 2007; Wei et al., 2012; Brand et al., 2013), the 15 members of Group I contained 27 WDs. Subgroup II genes exist early in the evolution of plants (Hori et al., 2014). Interestingly, *CsWRKY47* in Group IIa had unique sequences in the WD (WKKYGQK) except for *CsWRKY03* and *CsWRKY63* in Group I. The WD sequence variation may influence the normal interactions and binding specificities with downstream target genes (van Verk et al., 2008; Zhou et al., 2008). Recently, a great quantity of Group I and Group II functions have been identified. For example, *CitWRKY28* (Group I) and *CitNAC029* can promote the accumulation of cuticular wax in Arabidopsis leaves (Yang et al., 2022). In *Dioscorea composita*, the expression of *DcWRKY3* (Group I) is strongly affected by salt stress (Yu et al., 2022). The transcription level of *CsWRKY25* (Group I) is up-regulated in *Penicillium digitatum*-infected citrus peels (Wang et al., 2022b). *PcWRKY11* (Group II WRKY from *Polygonum cuspidatum*) significantly increases the tolerance to salt stress in transgenic *A. thaliana* (Wang et al., 2022a).

Compared with Group I and II WRKY TFs, Group III TFs alter C2H2 to C2HC zinc-finger motif C-X7-C-X26-HTC. It has been considered the most adaptable and most advanced in monocot evolution (Eulgem et al., 2000; Zhang and Wang, 2005). In addition, Group III is the youngest in the WRKY family (Song and Gao, 2014; Wu et al., 2016). Therefore, in numerous species, Group III is the key to determining the number of WRKY genes. Nevertheless, Group III WRKY genes are considered the most vital group in gene family evolution and seemingly play an important role in the adaptation and evolution of plants (Zhang and Wang, 2005; Goel et al., 2016; Shan et al., 2021; Khan et al., 2022). In *C. sinense*, 11 *CsWRKY* gene members belonged to Group III, and such a number is fewer than the corresponding number in most other plants, such as 13 in Arabidopsis (Kalde et al., 2003), 28 in rice (Wang et al., 2015), 35 in maize (Hu et al., 2021), 62 in *Dendrobium catenatum* (Zhang et al., 2022b), 57 in *Phalaenopsis*, and 43 in *Apostasia* (unpublished). These findings suggested the functional conservation of WRKY genes during orchid evolution.

We analyzed the function of *CsWRKY18* and indicated its role in the ABA pathway and abiotic stress. Different abiotic stresses can induce *AtWRKY30*. Meanwhile, the overexpression of *AtWRKY30* greatly enhances the resistance of Arabidopsis in response to salt stress (Scarpeci et al., 2013) and improves heat and drought stress tolerance in wheat (El-Esawi et al., 2019). Moreover, *AtWRKY30* expression is induced by oxidative stress treatment, fungal elicitor, mosaic virus, JA, auxin, SA, and ABA (Jiang et al., 2017; Zou et al., 2019). Evolutionary processes including duplication events and chromosomal set changes (polyploidy) could extend the members of a gene family in plants (Faraji et al., 2021; Heidari et al., 2021). Besides, it seems that WRKY gene family has been subjected to more evolutionary pressures and extended. However, evolutionary events could increase the members of gene family, but point mutations in coding site regions and upstream/downstream site of new members can affect the function of new members and cause diversity of members expression (Abdullah et al., 2021; Musavizadeh et al., 2021). Tandem duplication event as a 200 kb chromosome region containing two or more genes (Holub, 2001). All the 13 *CsWRKY* gene pairs among 64 WRKY genes were identified as segmental duplication, but no tandem duplication, indicating that tandem duplication events might have not articipated in the amplification of *CsWRKY* gene family. More functions of *CsWRKYs* should be further studied.

Furthermore, six *CsWRKYs* were subdivided into one subclass IIIa, and five *CsWRKYs* were classified into another subclass IIIb. *OsWRKY45* (Group IIIa) is an essential positive player in the resistance against the rice blast fungus *M. oryzae* (Shimono et al., 2007). Various abiotic stresses can induce *AtWRKY30* (Group IIIb). Meanwhile, the overexpression of *AtWRKY30* significantly improves the resistance of Arabidopsis in response to salt stress (Scarpeci et al., 2013). These years, WRKY-c311842 TFs play key roles in berberine accumulation in Coptis chinensis (Liu et al., 2022). As an essential plant hormone in plants, ABA plays a vital role in development, physiological processes, and stress responses, and the ABA signaling pathway is an important component of the stress regulation network (Li et al., 2017, 2018; Sun et al., 2018). Several WRKY TFs have been reported to be positive regulators of ABA-mediated stomatal closure, while some are negative regulators of seed germination and can also indirectly control flowering (Rushton et al., 2012). The ectopic expressions of *TaWRKY75*-A and *ZmWRKY79* in Arabidopsis improve the survival rate under drought stress by regulating ABA biosynthesis (Gulzar et al., 2021; Ye et al., 2021). *OsWRKY50* enhances salt stress tolerance *via* an ABA-independent pathway (Huang et al., 2021). In order to explore the *CsWRKY* Group III gene family, we elucidated the function of *CsWRKY18* in drought stress through regulating ABA biosynthesis.

In summary, we identified 64 WRKY genes in *C. sinense* genome and analyzed their expression patterns in response to GA, ABA, SA, IAA, JA, and ACC treatments and their expression profiles in the leaves of *C. sinense*. The ectopic expression of *CsWRKY18* in Arabidopsis improved the survival rate under drought stress, suggesting that *CsWRKY18* was a regulator in ABA biosynthesis.

## Data availability statement

Publicly available datasets were analyzed in this study. This data can be found at: NCBI, PRJNA743748.

## Author contributions

G-FZ and F-XY designed the experiments and edited the manuscript. Y-LW, J-PJ, and JG executed the experiments and assembled the figures. C-QL, DL, JL, and QX conducted the qRT-PCR and PPI. F-XY, J-PJ, and Y-LW wrote the paper with inputs from other authors. All authors contributed to the article and approved the submitted version.

## Funding

This research was funded by the Natural Science Foundation of Guangdong province (2017A030312004), grants from the National Key R&D Program (2018YFD1000404 and 2019YFD1001003), the National Natural Science Foundation of China (31902065), the Science and Technology Project of Guangdong Province (2019B030316033), Guangzhou Science and Technology Project (201707010307 and 202002030016), Innovation Team of Modern Agriculture Industry Technology System in Guangdong Province (2021KJ121), and Guangdong Academy of Agricultural Sciences Discipline Team Construction Project (202127TD and BZ202006).

## Conflict of interest

The authors declare that the research was conducted in the absence of any commercial or financial relationships that could be construed as a potential conflict of interest.

## Publisher’s note

All claims expressed in this article are solely those of the authors and do not necessarily represent those of their affiliated organizations, or those of the publisher, the editors and the reviewers. Any product that may be evaluated in this article, or claim that may be made by its manufacturer, is not guaranteed or endorsed by the publisher.

## Footnotes

^1^http://pfam.xfam.org/ (Accessed December 4, 2021).

^2^ http://hmmer.janelia.org/

^3^https://www.ncbi.nlm.nih.gov/Structure/cdd/ (Accessed December 3, 2021).

^4^http://web.expasy.org/protparam/ (Accessed January 3, 2022).

^5^ https://www.arabidopsis.org/

^6^http://rice.plantbiology.msu.edu/ (Accessed November 11, 2021).

^7^https://web.expasy.org/protparam/ (Accessed January 3, 2022).

^8^http://meme-suite.org/tools/meme (Accessed January 12, 2022).

^9^http://bioinformatics.psb.ugent.be/webtools/plantcare/html/ (Accessed February 2, 2022).

^10^ http://www.bork.embl.de/pal2nal/

^11^ https://wolfpsort.hgc.jp/

## Abbreviations

ABA: Abscisic acid
qRT-PCR: Quantitative RT-PCR
WGCNA: Weighted gene co-expression network analysis
WD: WRKY domain
GO: Gene ontology
PPI: Protein–protein interaction
ET: Ethylene
JA: Jasmonic acid
HMM: Hidden Markov Model
NJ: Neighbor-joining
Ks: Synonymous substitution rate
Ka: Non-synonymous substitution rate
