# Supplementary material for: Genome-wide identification of Cymbidium sinense WRKY gene family and the importance of its Group III members in response to abiotic stress
Source: Front Plant Sci. 2022 Jul 28;13:969010. doi: 10.3389/fpls.2022.969010 (PMC9365948; doi:10.3389/fpls.2022.969010)
Supplement: Supplementary file 1 [file Presentation_1.PPTX]

## Slide 1
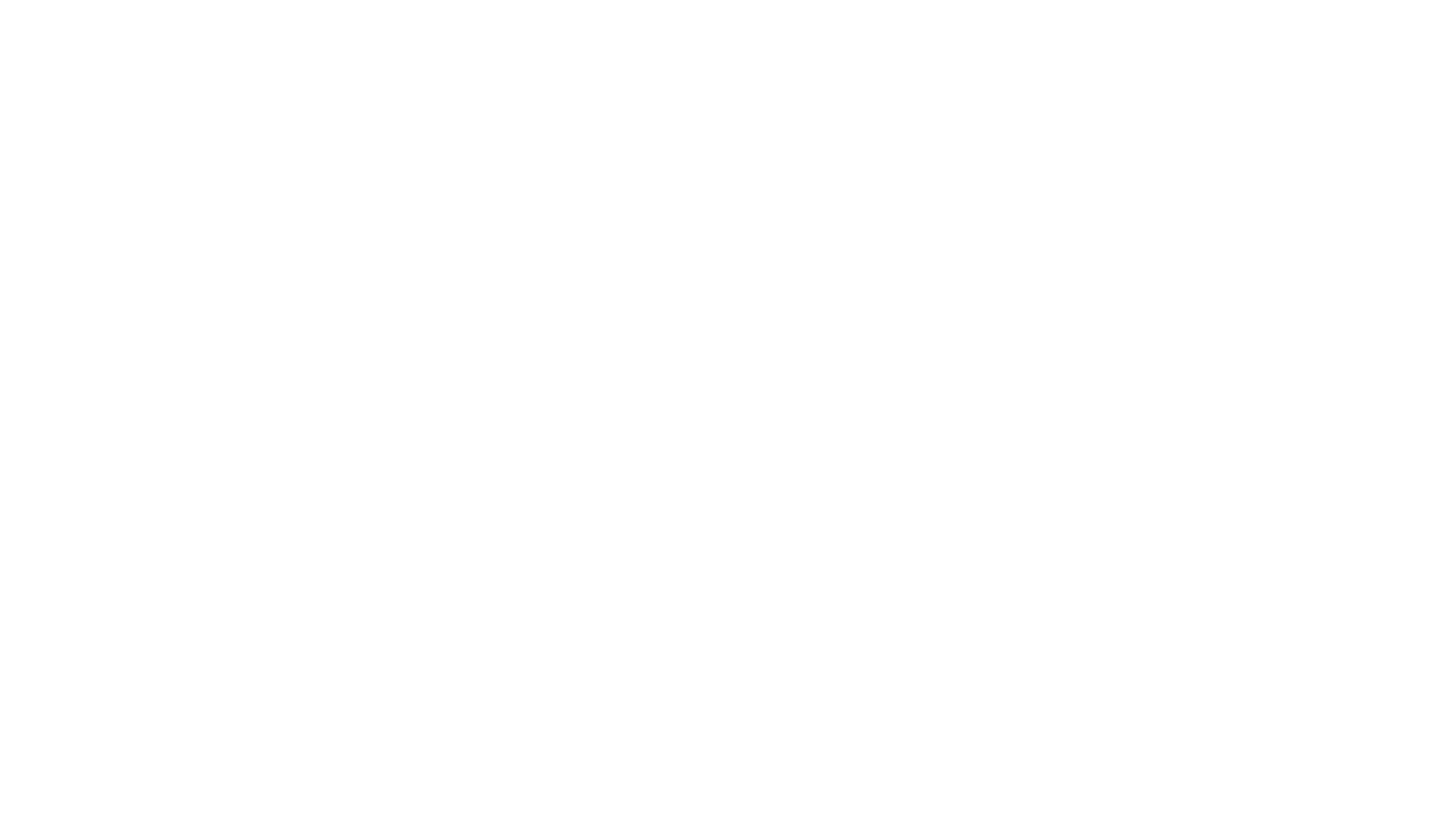

## Slide 2
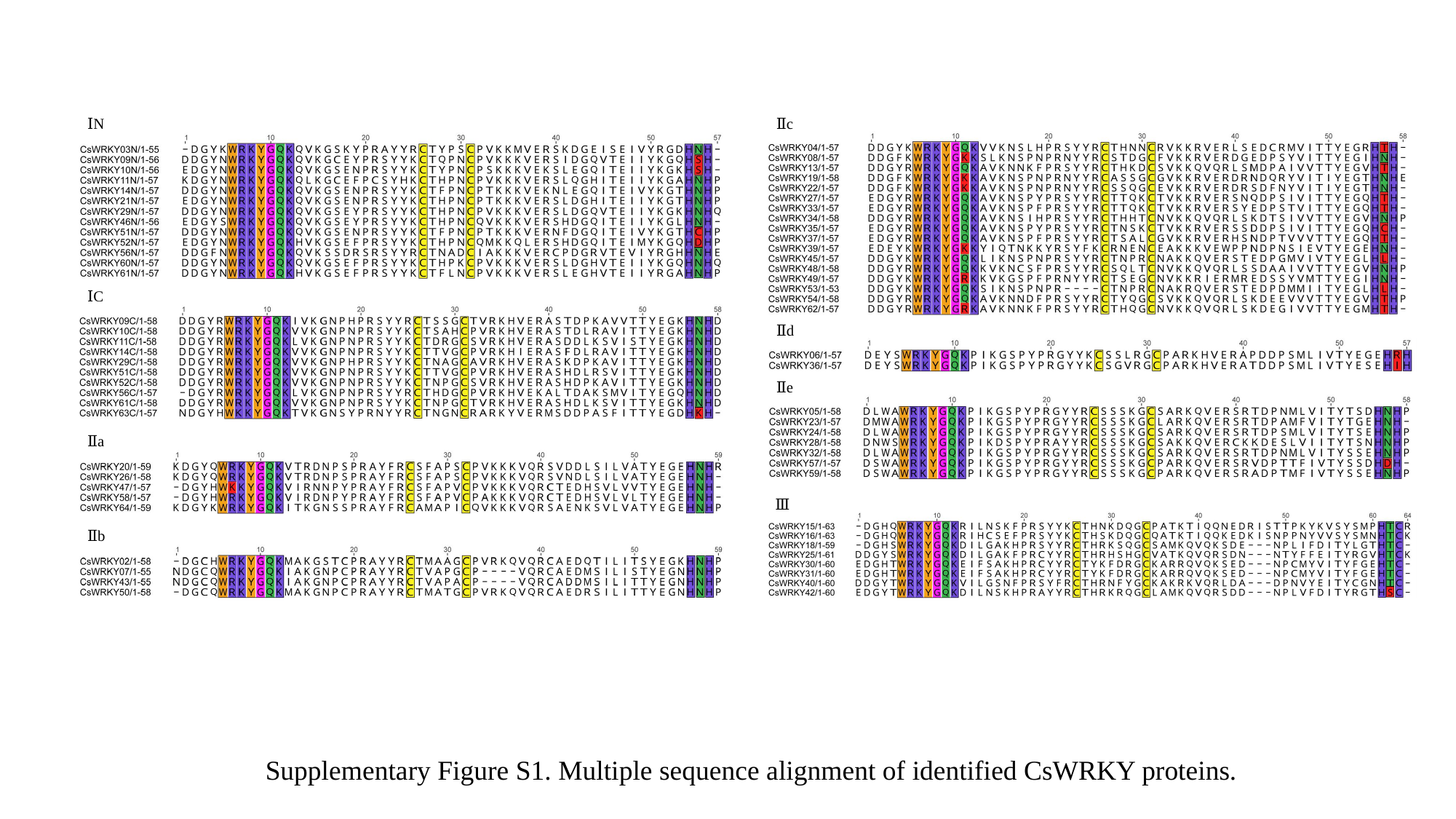

ⅠN
Ⅱc
ⅠC
Ⅱd
Ⅱe
Ⅱa
Ⅲ
Ⅱb
Supplementary Figure S1. Multiple sequence alignment of identified CsWRKY proteins.

## Slide 3
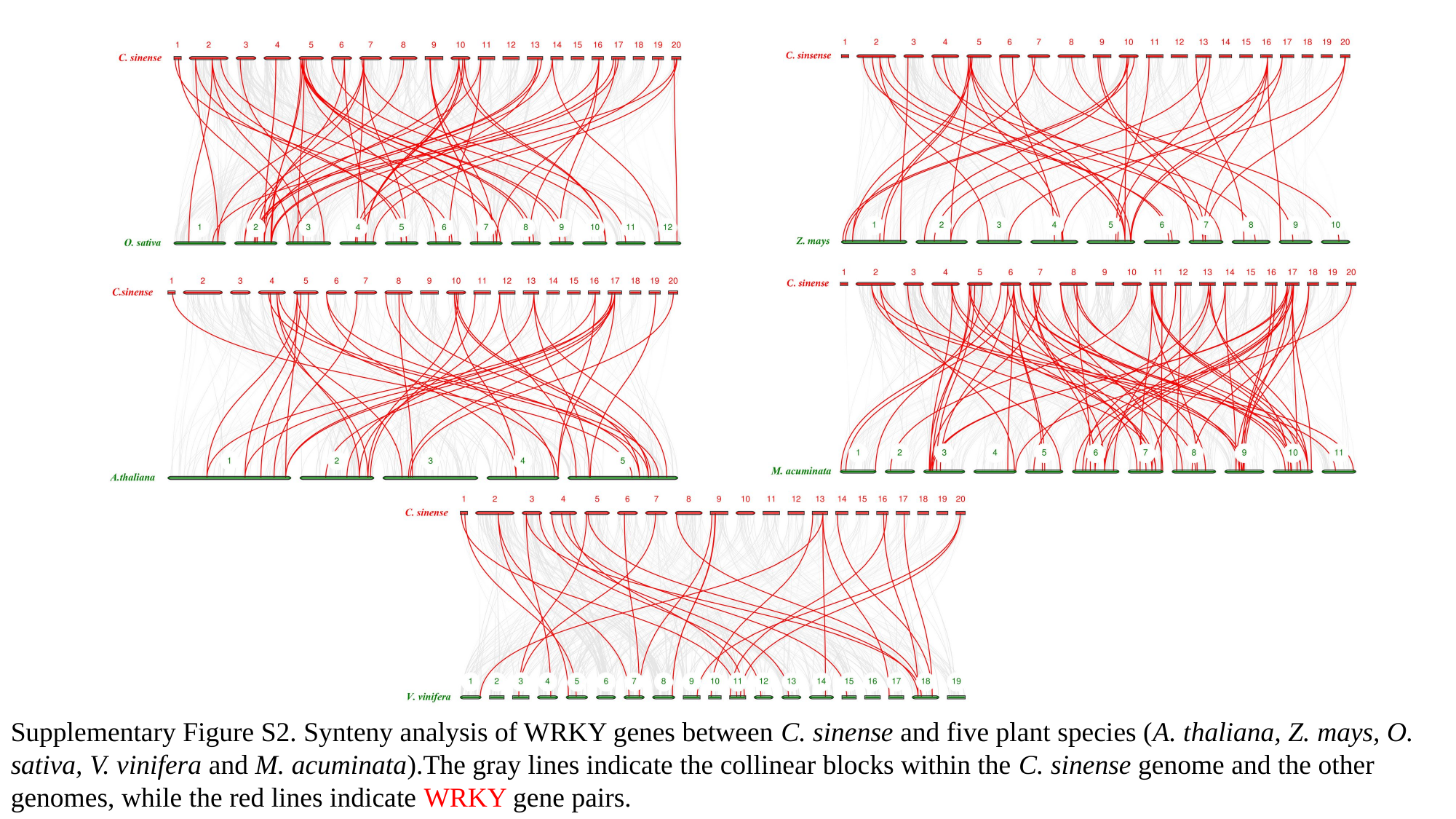

Supplementary Figure S2. Synteny analysis of WRKY genes between C. sinense and five plant species (A. thaliana, Z. mays, O. sativa, V. vinifera and M. acuminata).The gray lines indicate the collinear blocks within the C. sinense genome and the other genomes, while the red lines indicate WRKY gene pairs.

## Slide 4
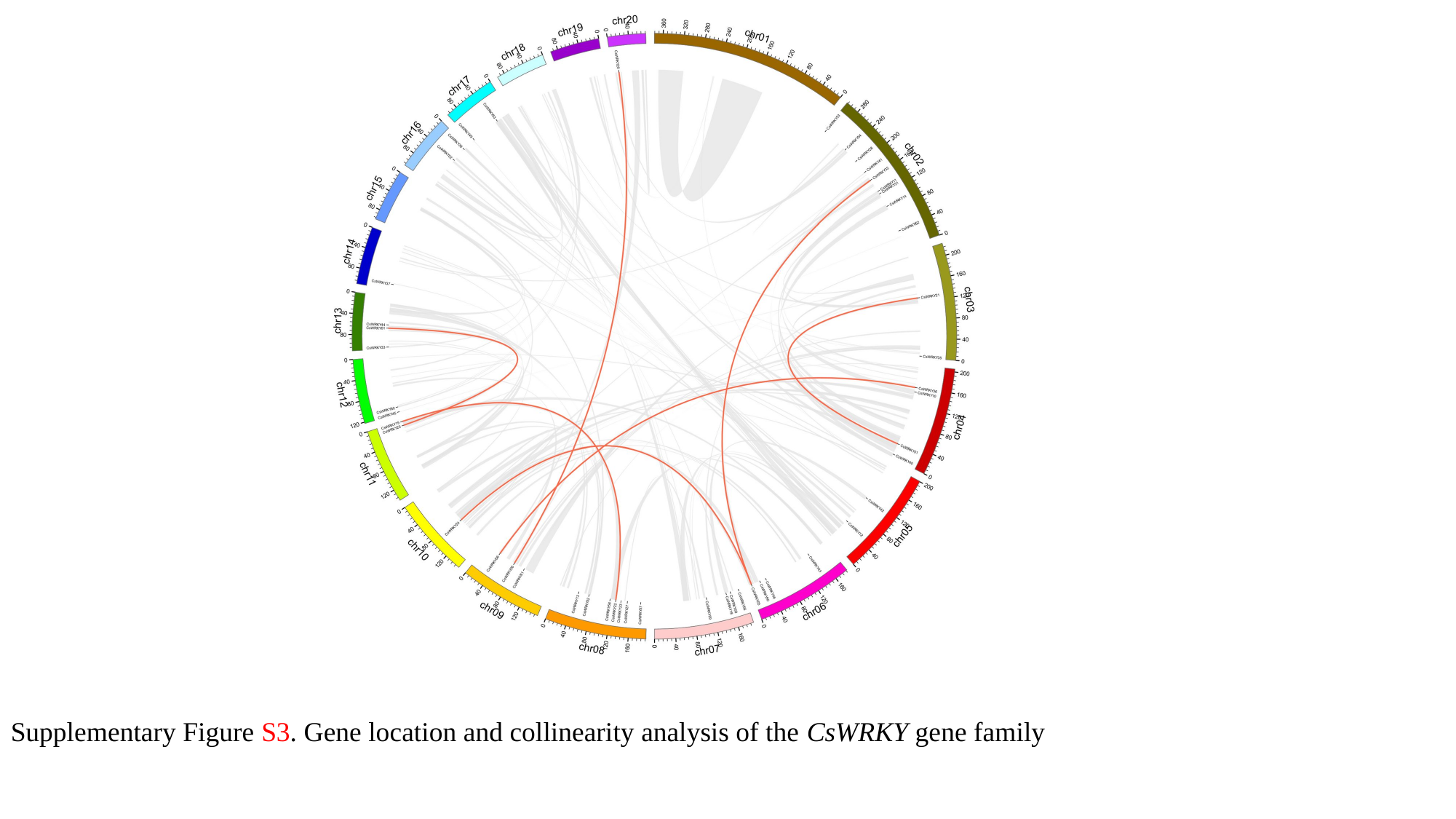

Supplementary Figure S3. Gene location and collinearity analysis of the CsWRKY gene family

## Slide 5
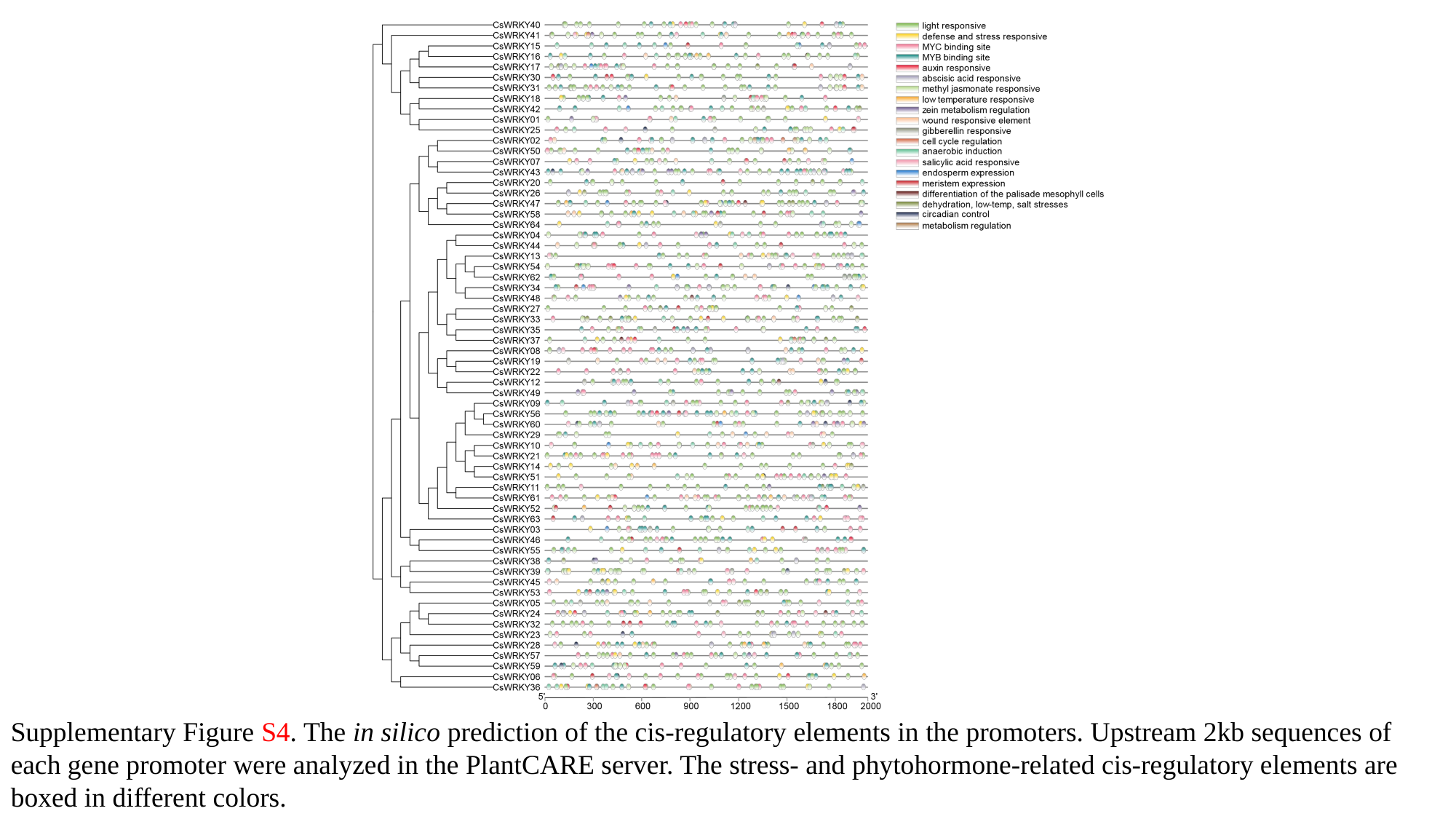

Supplementary Figure S4. The in silico prediction of the cis-regulatory elements in the promoters. Upstream 2kb sequences of each gene promoter were analyzed in the PlantCARE server. The stress- and phytohormone-related cis-regulatory elements are boxed in different colors.

## Slide 6
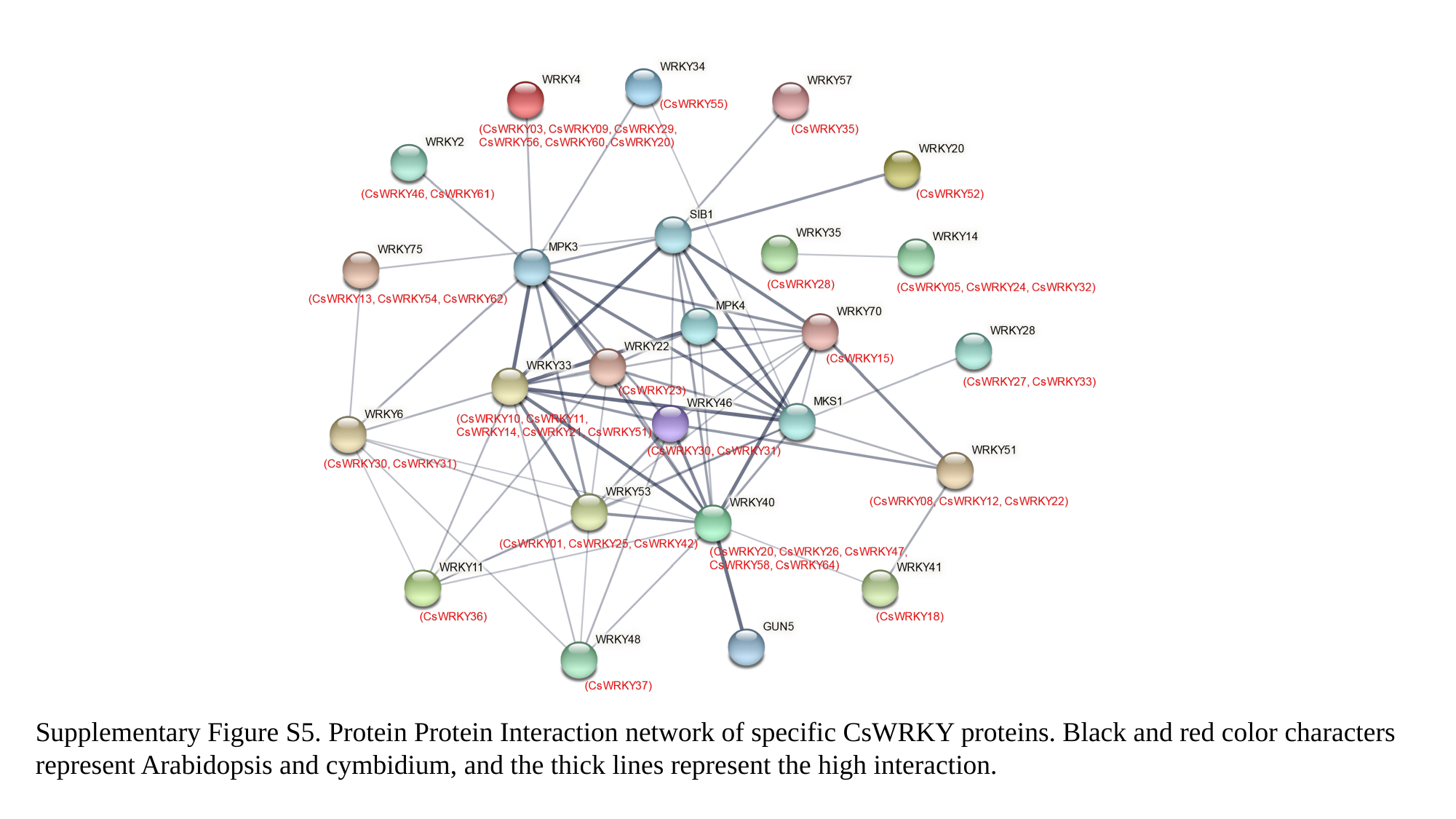

Supplementary Figure S5. Protein Protein Interaction network of specific CsWRKY proteins. Black and red color characters represent Arabidopsis and cymbidium, and the thick lines represent the high interaction.

## Slide 7
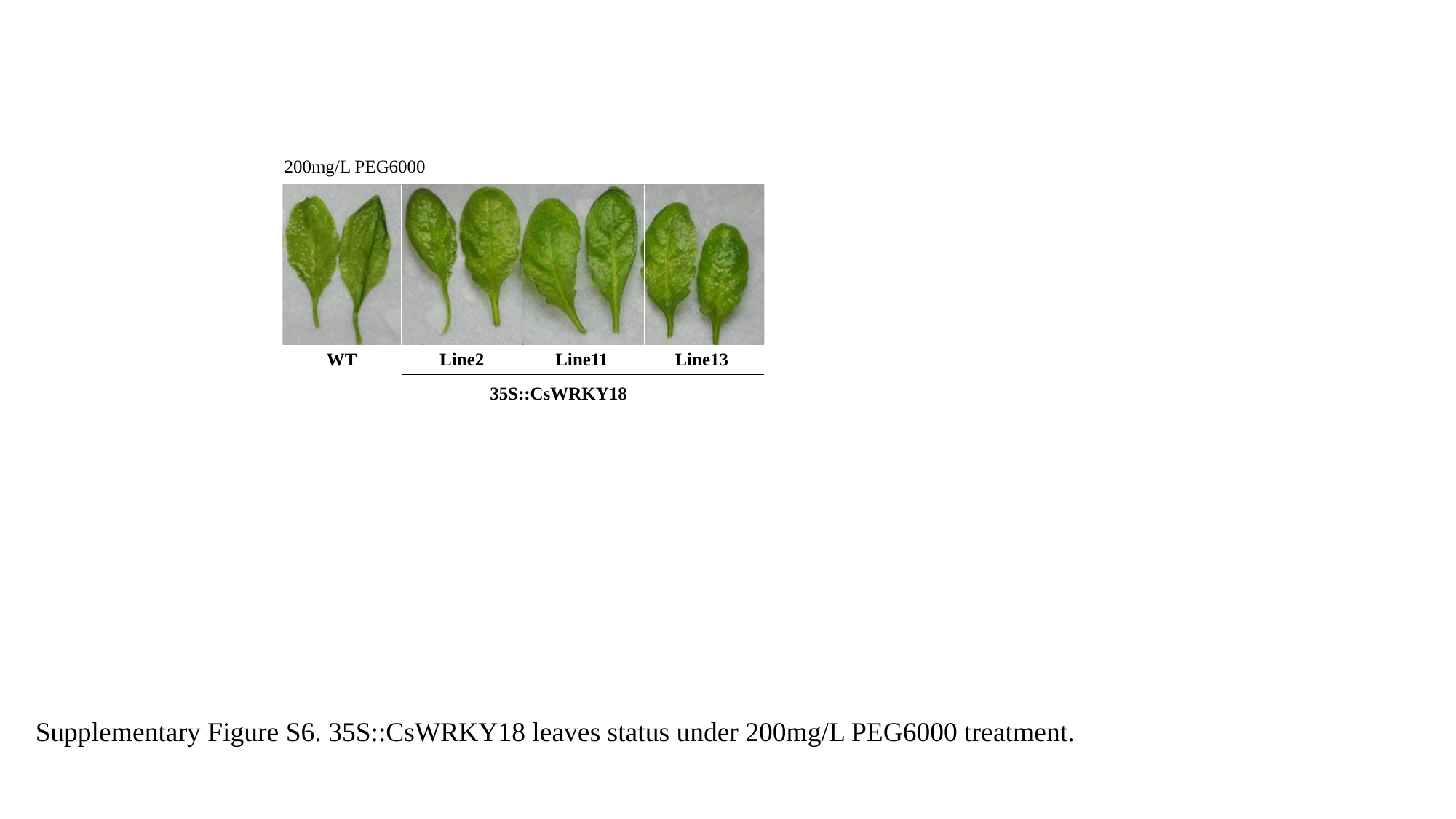

200mg/L PEG6000
WT
Line2
Line11
Line13
35S::CsWRKY18
Supplementary Figure S6. 35S::CsWRKY18 leaves status under 200mg/L PEG6000 treatment.

## Slide 8
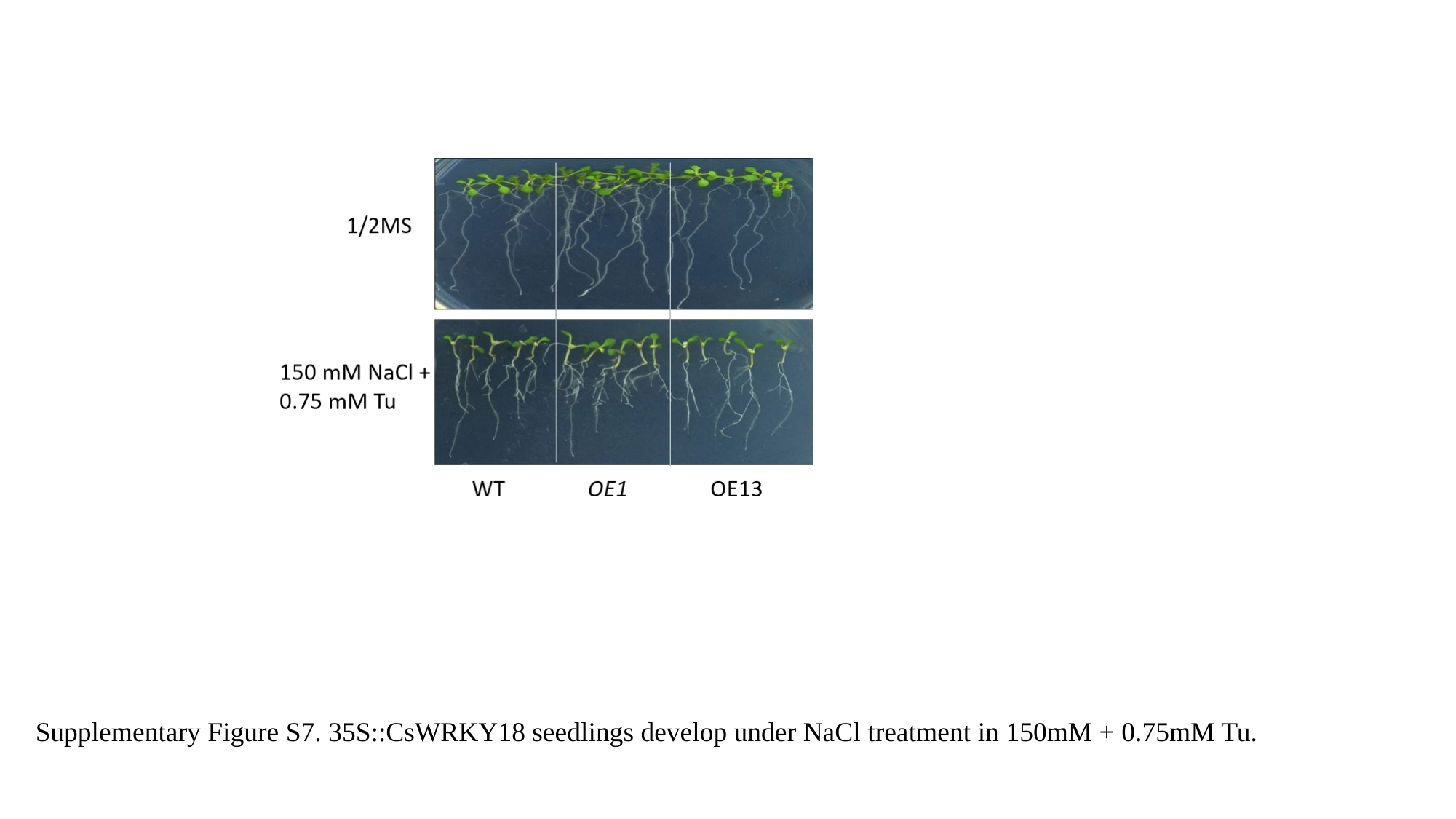

Supplementary Figure S7. 35S::CsWRKY18 seedlings develop under NaCl treatment in 150mM + 0.75mM Tu.
